# CircFAM13B promotes the proliferation of hepatocellular carcinoma by sponging miR-212, upregulating E2F5 expression and activating the P53 pathway

**DOI:** 10.1186/s12935-021-02120-6

**Published:** 2021-08-04

**Authors:** Ying Xie, Xiaofeng Hang, Wensheng Xu, Jing Gu, Yuanjing Zhang, Jianrong Wang, Xiucui Zhang, Xinghao Cao, Junjie Zhan, Junxue Wang, Jianhe Gan

**Affiliations:** 1 Department of Infectious Disease, The First Affiliated Hospital of Soochow University, 188 Shizi street, Suzhou, 215000 China; 2 Department of Infectious Disease, Changzheng Hospital, Naval Medical University, 415 Fengyang street, Shanghai, 200003 China

**Keywords:** circFAM13B, miR-212, E2F5, P53, HCC

## Abstract

**Background:**

Most of the biological functions of circular RNAs (circRNAs) and the potential underlying mechanisms in hepatocellular carcinoma (HCC) have not yet been discovered.

**Methods:**

In this study, using circRNA expression data from HCC tumor tissues and adjacent tissues from the Gene Expression Omnibus database, we identified out differentially expressed circRNAs and verified them by qRT-PCT. Functional experiments were performed to evaluate the effects of circFAM13B in HCC in vitro and in vivo.

**Results:**

We found that circFAM13B was the most significantly differentially expressed circRNA in HCC tissue. Subsequently, in vitro and in vivo studies also demonstrated that circFAM13B promoted the proliferation of HCC. Further studies revealed that circFAM13B, a sponge of miR-212, is involved in the regulation of E2F5 gene expression by competitively binding to miR-212, inhibits the activation of the P53 signalling pathway, and promotes the proliferation of HCC cells.

**Conclusions:**

Our findings revealed the mechanism underlying the regulatory role played by circFAM13B, miR-212 and E2F5 in HCC. This study provides a new theoretical basis and novel target for the clinical prevention and treatment of HCC.

**Supplementary Information:**

The online version contains supplementary material available at 10.1186/s12935-021-02120-6.

## Background

Hepatocellular carcinoma (HCC) has become one of the most common malignancies in the world and is the second most common cause of death in China [1, 2]. During the past decade, new drugs, such as sorafenib and immune checkpoint inhibitors, have significantly extended the survival of HCC patients. However, the prognosis for HCC patients remains poor. Thus, exploring the molecular mechanism of HCC is urgently needed to identify new diagnostic biomarkers and therapeutic targets.

Recently, accumulating evidence has demonstrated a close association between circular RNAs (circRNAs) and tumorigenesis in a variety of cancers [3]. The discoveries of circRNAs such as hsa_circ_0009361, circNEIL3, circRNA_000864, circPARD3 and circRNA_LARP4, provide new potential methods and approaches in the diagnosis, treatment and prognosis of colonic, cervical, pancreatic, throat and gastric cancers [4–8]. However, the roles played by circRNAs in HCC are rarely reported.

In this study, using the circRNA expression data from HCC tumor tissues and adjacent tissues provided by GSE97332 [9], we identified out differentially expressed circRNAs and verified them by qRT-PCT. We found that circFAM13B was the most significantly differentially expressed circRNA in HCC tissue. Next, we investigated the potential functions of circFAM13B and its underlying mechanism in the development of HCC. Further studies revealed that circFAM13B, a sponge of miR-212, upregulates E2F5 gene expression through competitive binding with miR-212, inhibites the activation of the P53 signalling pathway, and promoted the proliferation of HCC cells. Thus, circFAM13B may represent used as a novel biomarker and target for the diagnosis and clinical treatment of HCC.

## Methods

### HCC tissue collection and cell culture

Twenty HCC patients who did not receive preoperative radiotherapy or chemotherapy were recruited from May 2015 to July 2018 at Naval Medical University. Tumor and adjacent tissues were collected during surgery. All tissue samples were maintained in liquid nitrogen. All patients signed the informed consent form. All procedures and experiments of this study conformed to the Declaration of Helsinki, and were approved by the Ethics Committee of Naval Medical University.

HCC cell lines (HepG2, Smmc-7721, HLE, Huh7 and Bel-7404) and normal liver cells (LO2) were purchased from the Chinese Academy of Sciences Shanghai Institute of Life Sciences. Cells were cultured in high-glucose Dulbecco’s modified Eagle’s medium (DMEM) (Gibco, Vienna, Austria) containing 10% foetal bovine serum. Cells were incubated at 37 °C in 5% CO_2_ and 95% humidity. The circFAM13B plasmid, circFAM13B siRNA, sh-circFAM13B, E2F5 siRNA, controls, miR-NC, miR-212 mimic, and miR-212 inhibitor were all purchased from Tingzhou Bio (Shanghai, China).

### Quantitative reverse transcription polymerase chain reaction (qRT-PCR)

Total RNA was extracted using TRIzol™ reagent (Invitrogen™, Carlsbad, CA, USA). The cDNA of mRNAs were amplified with oligo (dT) primers and the cDNA of circRNA were amplified with random primers by the PrimeScript™ RT Master Mix reagent kit (TaKaRa, Otsu, Shiga, Japan). The obtained cDNAs were subjected qRT-PCR using SYBR® Premix Ex Taq™ (TaKaRa, Otsu, Shiga, Japan). Gene expression was normalized to GAPDH (for mRNAs) or U6 (for miRNAs) using the comparative cycle time (Ct) method (2^−ΔΔCt^). Each qRT-PCR was performed in triplicate and the mean values were calculated. Primer sequences are shown in Additional file 1: Table S1.

### Dual-luciferase reporter

The targeted binding sites of circFAM13B, E2F5 and miR-212 were generated by bioinformatic methods. CircFAM13B-WT and E2F5-WT luciferase plasmids containing wild-type binding sites and circFAM13B-MUT and E2F5-MUT plasmids containing mutated binding sites were constructed by Tingzhou Bio (Shanghai, China). The luciferase reporter plasmids were transfected with miR-NC or miR-212. The pCMV-AC-GFP-circFAM13B plasmid (Tingzhou Bio, Shanghai, China) and pcDNA3.0-p53 (Tingzhou Bio, Shanghai, China) were transfected with p21-luciferase reporter plasmid (Tingzhou Bio, Shanghai, China) into HepG2 and Smmc-7721 cells. Luciferase activities were measured using the Dual-Luciferase Reporter Assay System (Promega, Madison, Wisconsin, USA). Reporter assays were performed in triplicate.

### Cell counting kit-8 (CCK-8)

The proliferation of HCC cells was evaluated by the CCK-8 assay (Dojindo Laboratories, Kumamoto, Japan). Cells grown at logarithmic phase were inoculated into 96-well plates at a density of 5 × 10^4^ cells per well for 48 h at 37 °C with 5% CO_2_. 10 µl of CCK-8 was added in each well and further incubated for 2 h. OD values were detected under 450 nm by Synergy 4 (BioTek, Winooski, VT, USA). Each test was triplicated.

### Colony-formation assay

Cells were counted, diluted in DMEM (Gibco, Vienna, Austria)containing 10% FBS, plated into 6-well plates (500 cell/petri dish), and incubated for 2 weeks. The colony-forming densities were constantly monitored until the colonies were macroscopically observable. Colonies were rinsed with phosphate buffered saline (PBS), fixed in 4% paraformaldehyde (30 min), and stained with crystal-violet solution (2.5% in methanol). The stained cells were subsequently rinsed and dried. Finally, colony counting and imaging were performed.

### RNA immunoprecipitation (RIP)

A Magna RIP RNABinding Protein Immunoprecipitation Kit (Millipore, MA, USA) was used to investigate whether the ribonucleoprotein complex contained miRNA and its potential binding circRNA in HCC cells. Ago2 antibody (Millipore, Billerica, USA) and IgG (Millipore, Billerica, USA) were used for immunoprecipitation. The antibodies were added to cell lysates and rotated overnight. After incubating with proteinase K buffer for 30 min the next day, the immunoprecipitated RNAs were isolated and extracted using TRIzol™ reagent (Invitrogen™, Carlsbad, CA, USA). qRT-PCR was performed on the immunoprecipitated RNA. The relevant steps and reagents of qRT-PCR are the same as above.

### Fluorescence in situ hybridization (FISH)

To determine the subcellular location of circFAM13B and miR-212 in HepG2 cells, cells were fixed in 10% fixing solution in PBS for 5 min. Glass slides containing cell samples were dipped into fixing solution twice (10 min each), dehydrated in gradual concentrations of iced-cold ethanol solutions (70%, 90 and 100%), and dried. FISH wasperformed in a wet box containing 50% formamide and 50 ml 2× saline sodium citrate buffer at 37℃. The FITC-labelled circFAM13B probe and PE-labelled miR-212 probe were designed by Yansai Co. Ltd. (Shanghai, China). Sequences of the probes are listed in Additional file 1: Table S2.

### RNase R treatments

Total RNA of HepG2 cells was incubated for 30 min at 37 °C with 3 U/µg RNase R (Epicentre Technologies, Madison, USA), and subsequently the abundance of linear FAM13B RNA and circFAM13B RNA was analysed by qRT-PCR. The relevant steps and reagents of qRT-PCR are the same as above.

### Actinomycin D assay

HepG2 cells were exposed to 100 ng/ml actinomycin D (Sigma-Aldrich, St. Louis, USA) for 0 h, 4 h, 8 h, 12 h, 16 h, 20 h, and 24 h. Then, the cells were harvested, and total RNA was extracted. The stability of circFAM13B and FAM13B mRNA was analysed using qRT-PCR. The relevant steps and reagents of qRT-PCR are the same as above.

### Western blotting

Protein samples were prepared in RIPA lysis buffer (Thermo Scientific, Rockford, IL, USA). Aliquots of 30 µg protein were fractionated by SDS-PAGE and transferred to PVDF membranes (Merck Millipore, Schwalbach, Germany). The membranes were blocked in skim milk, rinsed in PBS and incubated with primary antibodies overnight [E2F5 1:1000 (Abcam, Cambridge, UK, ab59769), PUMA 1:1000 (Cell Signaling Technology, Danvers, MA, USA, 98672), P21 1:1000 (Cell Signaling Technology, Danvers, MA, USA, 2947), GAPDH 1:1000 (Cell Signaling Technology, Danvers, MA, USA, 5174), and β-Actin 1:1000 (Cell Signaling Technology, Danvers, MA, USA, 3700)]. The secondary antibodies, including goat anti-rabbit IgG (1:2000, Proteintech, USA, srbAF488-1) and goat anti-mouse IgG (1:2000, Proteintech, USA, SA00001-1), were then incubated with the membranes. The relative densities of the bands were quantified using densitometry (Quantity One software, BioRad).

### In vivo tumor growth

HepG2 cells were stable transfected with sh-circFAM13B and sh-NC lentivirus. Cells (10^7^) were subcutaneously injected under the right arm of each mouse. Ten female BALB/c nude mice were randomly grouped into sh-circFAM13B and sh-NC groups. Tumor growth was evaluated every 3 days after the injection. Mice were sacrificed 30 days after the injection using pentobarbital sodium (150 mg/kg) after the injection. The tumor tissue was collected and analysed.

### Statistical analysis

All statistical analyses were performed using SPSS 20 and GraphPad Prism 5. Data were subjected to independent-sample *t*-tests for comparisons between 2 groups, and one-way ANOVA for comparison among multiple groups. The significance level was *p* < 0.05 for all statistical analyses.

## Results

### Identification of circRNA differential expression and circFAM13B in HCC

**Fig. 1:**
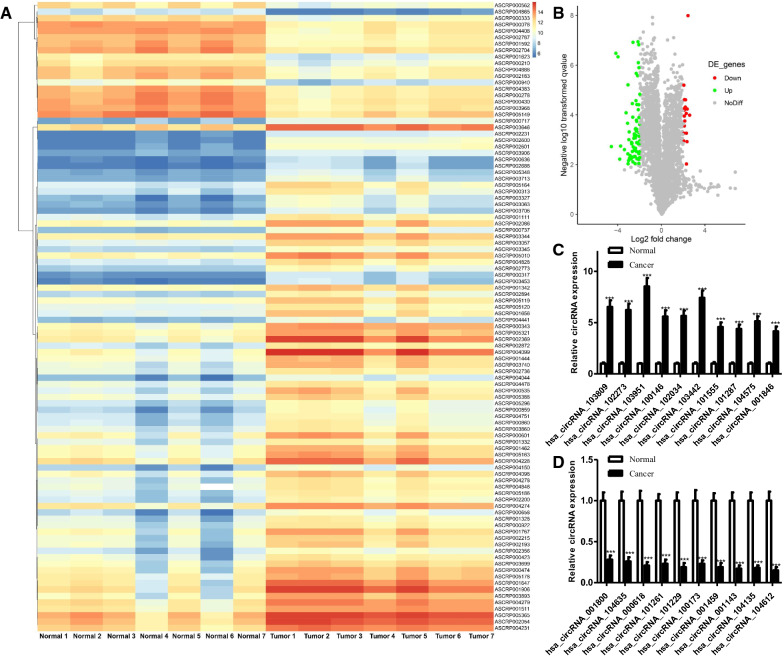
CircRNA differential expression in HCC. **A** Heatmap showed differentially expressed circRNAs based on 7 pairs of circRNA for HCC and adjacent tissues in GSE97332 (Fold Change > 4 and *P* < 0.01). **B** Volcano plots were constructed based on GSE97332 (Fold Change > 4 and *P* < 0.01) The red points represent differentially upregulated genes, and green points represent downregulated genes. **C**, **D** Validation of circRNAs in tumor tissues and matched para-carcinoma tissues by qRT-PCR (***P < 0.001, **P < 0.01, *P < 0.05)

**Fig. 2:**
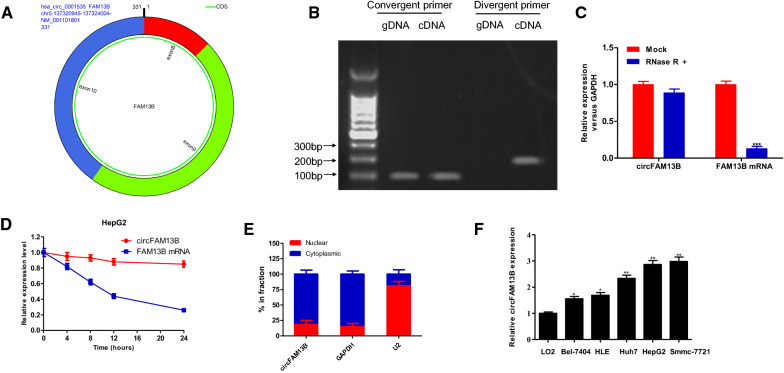
Identification of circRNA differential expression and circFAM13B in HCC. **A** The exonic information of circFAM13B was illustrated as indicated. **B** qRT-PCR products with divergent primers showing circularization of circFAM13B. **C** qRT-PCR analysis for the expression of circFAM13B and FAM13B mRNA after treatment with RNase R in HepG2. **D** qRT-PCR analysis for the expression of circFAM13B and FAM13B mRNA after treatment with Actinomycin D in HepG2. **E** Nuclear-cytoplasmic fractionation experiment identified the subcellular location of circFAM13B. **F** The expression level of circFAM13B in in normal liver cell and HCC cells was determined by qRT-PCR (***P < 0.001, **P < 0.01, *P < 0.05)

The GSE97332 database [9] was used to investigate the differential expression of circRNAs between HCC tissues and adjacent tissues. GSE97332 includes 7 pairs of circRNA samples for HCC and adjacent tissue. The results were further screened using the criteria of fold change > 4 and *P* < 0.01. In the results, 98 differentially expressed circRNAs, of which 80 were upregulated and 18 were downregulated in HCC, were identified (Fig. 1A, B). We next verified the top 10 up- and down-regulated differentially expressed circRNAs by qRT-PCR. As shown in Fig. 1C, D, the most significance of the differentially expressed hsa_circRNA_103951 was confirmed. Hsa_circRNA_103951 is also referred to as hsa_circ_0001535 according to the circBase database (www.circbase.org). It is formed by the cyclization of the 8th to 10th exon of FAM13B, with a spliced length of 331 bp and is referred to as circFAM13B hereafter (Fig. 2A). To confirm whether circFAM13B was formed by head-to-end splicing, divergent primers and convergent primers were designed for the amplification of the circular transcript of circFAM13B. The circular structure of circFAM13B was confirmed by electrophoretic testing of nucleic acids (Fig. 2B). Additionally, as revealed by the RNase R test, the line structure of FAM13B was digested by exonuclease, while the circular structure of circFAM13B was not digested by exonuclease (Fig. 2C). Actinomycin D assay showed the half-life of circFAM13B was significantly higher than that of lineal FAM13B (Fig. 2D). Using nuclear and cytoplasmic protein extraction, we identified the subcellular localization of circFAM13B. In the results, circFAM13B was found to be primarily localized in the cytoplasm of HCC (Fig. 2E). We also investigated the circFAM13B expression level in cells. In the results, circFAM13B was found to be significantly more highly expressed in HCC cell lines than in normal liver cells (Fig. 2F).

### CircFAM13B promotes proliferation of HCC

**Fig. 3:**
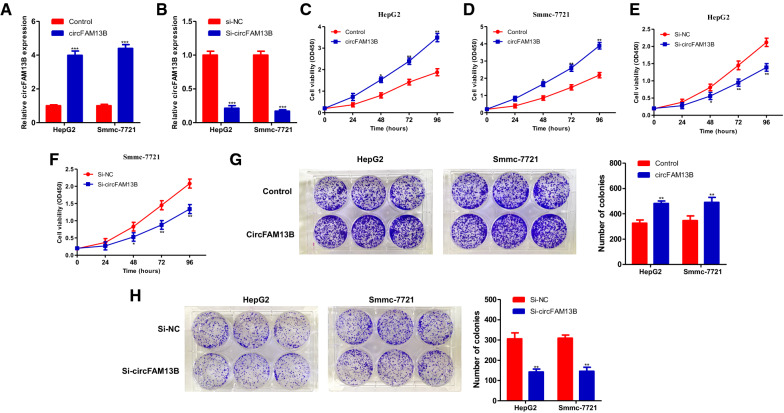
circFAM13B promotes proliferation of HCC in vitro. **A**, **B** Verification efficiency of overexpressed circFAM13B or down-expressed circFAM13B in HepG2 and Smmc-7721 by qRT-PCR. **C**, **D** CCK-8 assays were performed to determine the cell proliferation of HepG2 and Smmc-7721 transfected with overexpression plasmid (circFAM13B) or control vector. **E**, **F** CCK-8 assays were performed to determine the cell proliferation of HepG2 and Smmc-7721 transfected with downexpression siRNA (si-circFAM13B) or si-NC. **G** Colony-formation assays were performed to determine the cell proliferation of HepG2 and Smmc-7721 transfected with overexpression plasmid (circFAM13B) or control vector. **H** Colony-formation assays were performed to determine the cell proliferation of HepG2 and Smmc-7721 transfected with downexpression siRNA (si-circFAM13B) or si-NC (***P < 0.001, **P < 0.01, *P < 0.05)

To investigate the biological roles played by circFAM13B in HCC, both a circFAM13B overexpression plasmid and a circFAM13B RNAi plasmid were constructed. Both plasmids were expressed in HepG2 and Smmc-7721 cells, and the efficiencies of overexpression and interference were evaluated using qRT-PCR (Fig. 3A, B). As revealed by the CCK-8 test, increased expression of circFAM13B significantly promoted the proliferation of HCC, while decreased expression of circFAM13B significantly inhibited the proliferation of HCC (Fig. 3C–F). These results were further supported by the results of the colony-formation assay (Fig. 3G, H).

### CircFAM13B promotes tumor growth in vivo

**Fig. 4:**
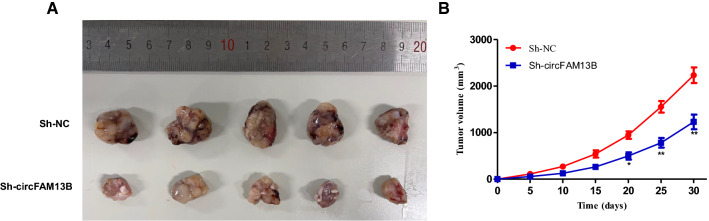
circFAM13B promotes proliferation of HCC in vivo. **A** Tumors collected of sh-NC and sh-circFAM13B for 30 days in vivo. **B** Growth curves of sh-NC and sh-circFAM13B (***P < 0.001, **P < 0.01, *P < 0.05)

To investigate the functions of circFAM13B in vivo, HepG2 cells transfected with circFAM13B RNAi or control plasmid were subcutaneously injected into female nude mice. In the results, we found that interfering with circFAM13B expression significantly inhibited tumor growth, as revealed by significantly smaller-sized tumors in mice that received injection of HepG2 cells with circFAM13B RNAi than in the controls (Fig. 4A, B).

### CircFAM13B is a molecular sponge of miR-212-3p

**Fig. 5:**
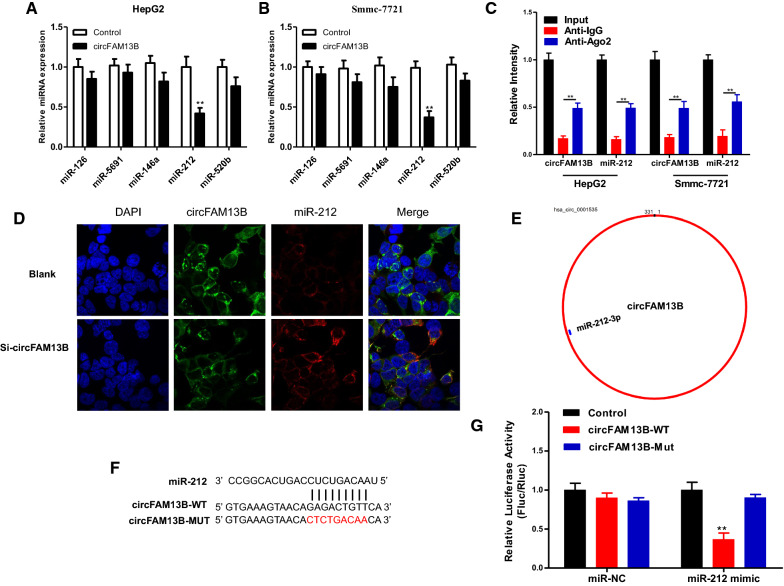
circFAM13B is the molecular sponge of miR-212-3p. **A**, **B** qRT-PCR assays were performed to determine the expression level of miR-126, miR-5691, miR-146a, miR-212 and miR-520b in HepG2 and Smmc-7721 transfected with overexpression plasmid (circFAM13B) or control vector. **C** The co-precipitated miR-212 and circFAM13B were subjected to qRT-PCR by RIP experiments in HepG2 and Smmc-7721. **D** FISH showed that circFAM13B and miR-212 were co-localized in the cytoplasm of HepG2. **E**, **F** Schematic illustration of circFAM13B-wt and circFAM13B-mut luciferase reporter vectors. **G** The relative luciferase activities were detected in HepG2 transfected with circFAM13B-wt or circFAM13B-mut and miR-212 mimics or miR-NC (***P < 0.001, **P < 0.01, *P < 0.05)

To investigate the molecular mechanism associated with the function of circFAM13B, we predicted miRNAs with potential binding sites on circFAM13B using circMir software (http://www.bioinf.com.cn/?page_id=10#comments). CircMir software predicts the binding sites based on the databases miRanda 2010 Release (http://www.microrna.org/microrna/getDownloads.do) and RNAhybrid-2.1.2 (https://bibiserv.cebitec.uni-bielefeld.de/rnahybrid/). In the results, the top 5 most likely miRNAs were predicted to be miR-126-5p, miR-5691, miR-146a-3p, miR-212-3p and miR-520b-3p. These miRNAs demonstrated binding and interacting potentials to circFAM13B. Of these miRNAs detected in HepG2 and Smmc-7721 cells overexpressing circFAM13B, miR-212 expression was significantly reduced (Fig. 5A, B). As revealed by the RIP results in both HepG2 and Smmc-7721 cells, circFAM13B and miR-212 were effectively enriched and downregulated by the Ago2 antibody (Fig. 5C). Using FISH, circFAM13B and miR-212 were found to be colocalized in HepG2 cells. We found that, circFAM13B was primarily distributed in the cytoplasm of cells, and its expression was negatively correlated with the expression of miR-212 (Fig. 5D). Subsequently, the binding of circFAM13B and miR-212 was evaluated using the dual-luciferase reporter assay. CircFAM13B-WT and circFAM13B-MUT were constructed (Fig. 5E, F). In the results, co-transfection of miR-212 mimic and circFAM13B-WT plasmids significantly reduced the fluorescence signal. Meanwhile, co-transfection of miR-212 mimic and circFAM13B-MUT plasmids did not affect the fluorescence signal (Fig. 5G). Collectively, the above results showed that circFAM13B plays as a molecular sponge of miR-212 in HCC.

**Fig. 6:**
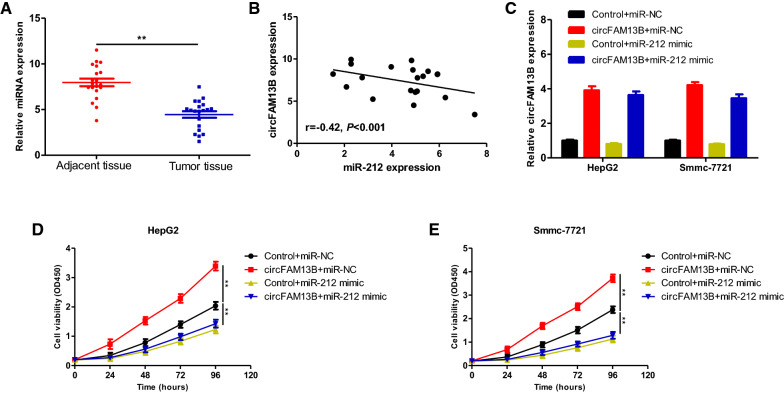
**A** qRT-PCR assays were performed to determine the expression level of miR-212 in the 20 cases of HCC tissue and adjacent tissue. **B** The expression of circFAM13B was negatively correlated with the expression of miR-212 in the 20 cases of HCC tissues. **C** qRT-PCR assays were performed to determine the expression level of circFAM13B in HepG2 and Smmc-7721 transfected with Control + miR-NC, circFAM13B + miR-NC, Control + miR-212 mimic or circFAM13B + miR-212 mimic. **D**, **E** CCK-8 assays were performed to determine the cell proliferation of HepG2 and Smmc-7721 transfected with Control + miR-NC, circFAM13B + miR-NC, Control + miR-212 mimic or circFAM13B + miR-212 mimic (***P < 0.001, **P < 0.01, *P < 0.05)

Previous studies have indicated that miR-212 acts as a tumor suppressor gene in HCC [10–12]. As verified by qRT-PCR analysis of miR-212 expression in the 20 cases of HCC tissue and their respective adjacent tissue, miR-212 was significantly lower expressed in HCC tissue (Fig. 6A). We also found that the expression of circFAM13B was negatively correlated with the expression of miR-212 in the 20 HCC tissues (Fig. 6B). To explore whether circFAM13B played biological roles in the regulation of miR-212, rescue experiments were conducted. Transfection of HepG2 and Smmc-7721 cells was conducted as follows: (1) Control + miR-NC, (2) circFAM13B + miR-NC, (3) Control + miR-212 mimic, and (4) circFAM13B + miR-212 mimic. The expression of circFAM13B after transfection in 4 group was showed in Fig. 6C. In the results, the miR-212 mimic significantly reduced the cell proliferation effects caused by upregulated circFAM13B (Fig. 6D, E), indicating that circFAM13B potentially promotes the proliferation of HCC via the regulation of miR-212.

### CircFAM13B promotes E2F5 expression, activates the ERK signalling pathway and upregulates the proliferation of HCC via absorption of miR-212

**Fig. 7:**
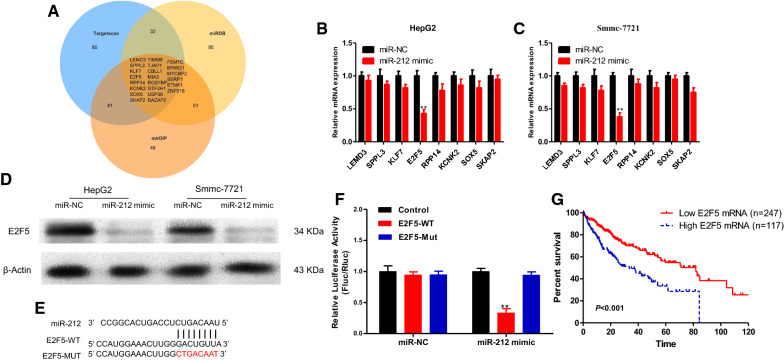
**A** TargetScan, miRDB and mirDIP were used to predict the potential target genes of miR-212. **B**, **C** qRT-PCR assays were performed to determine the expression level of LEMD3, TIMM9, SPPL3, KLF7, E2F5, LEMD3, RPP14, KCNK2, SOX5 and SKAP2 in HepG2 and Smmc-7721 transfected with miR-NC or miR-212 mimic. **D** Western blotting were performed to determine the expression level of E2F5 in HepG2 and Smmc-7721 transfected with miR-NC or miR-212 mimic. **E** Schematic illustration of E2F5-wt and E2F5-mut luciferase reporter vectors. **F** The relative luciferase activities were detected in HepG2 transfected with E2F5-wt or E2F5-mut and miR-212 mimics or miR-NC. **G** According to Kaplan–Meier Plotter, HCC patients with high mRNA expression level of E2F5 demonstrated poorer prognosis than HCC patients with relatively low mRNA expression level of E2F5 (***P < 0.001, **P < 0.01, *P < 0.05)

To identify possible targets of miR-212, we predicted the top 200 target genes of miR-212 using three databases TargetScan, miRDB and mirDIP, respectively, and take the intersection of them to obtain 22 potential target genes (LEMD3, SPPL3, KLF7, E2F5, RPP14, KCNK2, SOX5, SKAP2, TIMM9, TJAP1, CBLL1, MIA3, RGS7BP, GTF2H1, USP38, DAZAP2, FEM1C, BRWD1, MYCBP2, SERP1, ETNK1, ZNF516) (Fig. 7A). Through a literature review, 8 genes: LEMD3, SPPL3, KLF7, E2F5, RPP14, KCNK2, SOX5 and SKAP2, were closely related to the process of tumorigenesis.

We subsequently validated these 8 genes as potential target genes of miR-212 in both HepG2 and Smmc-7721 cell lines with upregulated expression of miR-212. In the results, we found that transcription levels of E2F5 were significantly reduced as revealed by qRT-PCR (Fig. 7B, C); meanwhile, protein levels of E2F5 were significantly decreased, as revealed by western blotting (Fig. 7D). Subsequently, E2F5 3′ UTR-WT and E2F5 3′UTR-MUT dual-luciferase reporter gene plasmids in combination with miR-212 were constructed for dual fluorescence enzyme reporting gene experiments (Fig. 7E). In the results, the miR-212 mimic significantly reduced the fluorescence signal of E2F5 3′ UTR-WT, while the fluorescence signal of E2F5 3′UTR-MUT was not affected (Fig. 7F), suggesting that E2F5 is a direct target gene of miR-212. According to Kaplan–Meier Plotter (http://kmplot.com/analysis/index.php), HCC patients with high transcriptional levels of E2F5 had poorer prognosis than HCC patients with relatively low transcriptional levels of E2F5 (Fig. 7G).

**Fig. 8:**
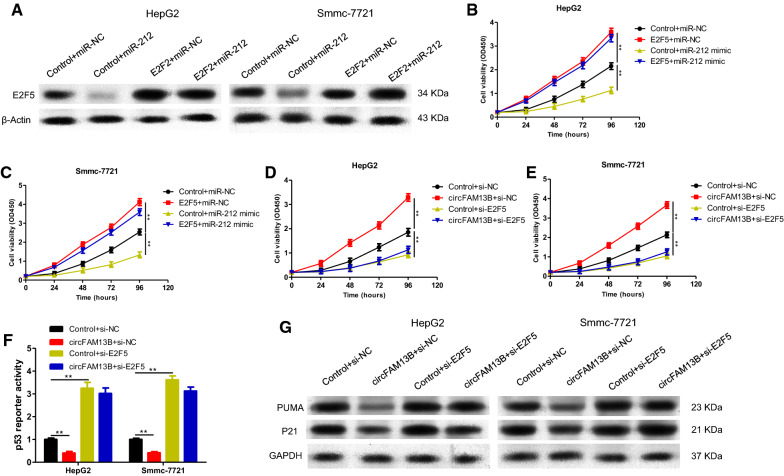
**A** Western blotting were performed to determine the protein level of E2F5 in HepG2 and Smmc-7721 transfected with Control + miR-NC, E2F5 + miR-NC, Control + miR-212 mimic and E2F5 + miR-212 mimic. **B**,** C** CCK-8 assays were performed to determine the cell proliferation of HepG2 and Smmc-7721 transfected with Control + miR-NC, E2F5 + miR-NC, Control + miR-212 mimic and E2F5 + miR-212 mimic. **D**, **E** CCK-8 assays were performed to determine the cell proliferation of HepG2 and Smmc-7721 transfected with Control + si-NC, circFAM13B + si-NC, Control + si-E2F5, circFAM13B + si-E2F5. **F** The p53 luciferase reporter were detected in HepG2 and Smmc-7721 transfected with Control + si-NC, circFAM13B + si-NC, Control + si-E2F5, circFAM13B + si-E2F5. **G** Western blotting were performed to determine the protein level of PUMA and P21 in HepG2 and Smmc-7721 transfected with Control + si-NC, circFAM13B + si-NC, Control + si-E2F5, circFAM13B + si-E2F5 (***P < 0.001, **P < 0.01, *P < 0.05)

The roles of E2F5 in HCC as an oncogene have been previously reported [13–15]. To investigate whether miR-212 plays a biological role by regulation of E2F5, rescue experiments were conducted. Both HepG2 and Smmc-7721 cells were transfected as follows: (1) Control + miR-NC, (2) E2F5 + miR-NC, (3) Control + miR-212 mimic, and (4) E2F5 + miR-212 mimic. Protein levels of E2F5 after transfection were verified by western blotting (Fig. 8A). In the results, in E2F5 overexpressing cells, upregulated miR-212 did not rescue the increase in HCC proliferation caused by E2F5 (Fig. 8B, C), demonstrating that miR-212 promotes the proliferation of HCC through E2F5.

To determine whether circFAM13B regulates the proliferation of HCC via E2F5, rescue experiments were conducted. Both HepG2 and Smmc-7721 cells were transfected as follows: (1) Control + si-NC, (2) circFAM13B + si-NC, (3) Control + si-E2F5, and (4) circFAM13B + si-E2F5. As revealed by the CCK-8 proliferation experiment, overexpression of circFAM13B did not affect the proliferation of E2F5 knockout cells (Fig. 8D, E). The activity of the p53 signalling pathway was also assessed using a p21-luciferase reporter assay. Overexpression of circFAM13B inhibited the activity of the p53 signaling pathway (Fig. 8F). However, in cells with inhibited expression of E2F5, overexpression of circFAM13B did not affect the activity of the p53 signalling pathway (Fig. 8F). Meanwhile the protein levels of key molecules of the p53 signalling pathway, PUMA and P21, show the same result (Fig. 8G). Collectively, these findings demonstrate that circFAM13B acts as an oncogene in the development of HCC, and circFAM13B function as a ceRNA by competitively binding to miR-212, upregulates expression of E2F5, inhibits the p53 signalling pathway, and promotes the proliferation of HCC.

## Discussion

CircRNAs are endogenous non-coding RNAs that have attracted great attention of researchers in recent years. Recent new evidence suggests that circRNAs act as molecular sponges of miRNAs, transcriptional regulators and protein encoding genes and play important roles in tumor development [3]. However, only a limited number of circRNAs have been well described until now. In this study, we found that circFAM13B was significantly increased in HCC tissue and cells. Subsequently, in vitro and in vivo studies also demonstrated that circFAM13B promotes the proliferation of HCC cells, while circFAM13B knockout inhibits proliferation of HCC cells. Our findings provide evidence that circFAM13B is an important regulator in the development of HCC.

The ceRNA hypothesis proposes that the RNA transcription of mRNAs, lncRNAs, pseudogenes and circRNAs forms a new complex regulatory network and mechanism at the posttranscriptional level for intermodulation by competing and sharing MREs. Growing evidence suggests that several circRNAs act as molecular sponges for miRNAs. For example, hsa_circ_0009361 acts as a ceRNA of miR-582 and inhibits the proliferation and metastasis of colorectal cancer cells [4]. In cervical cancer, circNEIL3 promotes tumorigenesis through the regulation of KLF12 by acting as a ceRNA of miR-137 [5]. Additionally, studies have demonstrated that cyclic circRNA_000864 absorbs miR-361, relieves the inhibitory effects on the target gene BTG2 in the pancreatic cancer, and promoted tumor proliferation and tumorigenesis [6]. Moreover, circLARP4 inhibited the development and metastasis of gastric cancer cells by targeting the miR-424/LATS1 axis [8]. In HCC, ceRNA is formed by hsa_circRNA_104348, miR-187-3p and RTKN2 and regulates the proliferation of HCC cells [16]. In this study, using bioinformatics approaches, we predicted the potential binding sites between circFAM13B and miR-212. Using FISH, we found that circFAM13 and miR-212 are localized in the cytoplasm. We further confirmed the direct interaction between circFAM13 and miR-212. Therefore, we proposed that circFAM13 potentially acts as an oncogenic gene in HCC via the adsorption of miR-212 by functioning as a molecular sponge.

Previous studies have provided evidence for the roles played by miR-212 as a tumor suppressor gene in different types of cancer [10–12]. MiR-212 was found to be significantly reduced in gastric cancer tissue. MiR-212 was also demonstrated to inhibit the proliferation of gastric cancer cells through the inhibition of MeCP2 protein expression [17]. In HCC, miR-212 inhibited the proliferation of HCC through regulation of the RBP2/CDKI signalling pathway [18]. In cervical cancer, miR-212 inhibited the proliferation and metastasis of cervical cancer cells by inhibiting TCF7L2 [19]. In this study, we found that miR-212 exhibits reduced expression in HCC tissue. Further evidenced by rescue experiments, we found that circFAM13B promotes the proliferation of HCC through the regulation of miR-212.

According to the ceRNA hypothesis, circRNA competitively bind to miRNAs to increase the expression of target genes. Through bioinformatics analysis, E2F5 was demonstrated to be a potential target gene for miR-212. Previous studies have demonstrated the oncogenic effect of E2F5 in different types of cancer [20–22], including HCC [13–15]. E2F5 was also reported to inhibit p53 and promote the proliferation and invasiveness of tumor cells in in malignant tumors [23]. In our results, as revealed by the dual-luciferase reporter gene, miR-212 potentially targets the 3′-UTR of E2F5. Further demonstrated by the rescue experiment, E2F5 was a direct target gene of miR-212. MiR-212 promoted the proliferation of HCC cells through the regulation of E2F5 expression. For further validation of the interaction between circFAM13B and E2F5, we found that circFAM13B upregulates the expression of E2F5 and inhibits the p53 signalling pathway. Moreover, circFAM13B was unable to regulate proliferation in E2F5 knockout cells. Our results demonstrated that circFAM13B acts as a molecular sponge for miRNA-212 and relieves the inhibitory effect of miR-212 on the target gene E2F5 in HCC.

Collectively, we found that through the absorption of miR-212, circFAM13B upregulates the expression of E2F5, activated ERK signaling pathway, and promotes the proliferation of HCC cells. Our findings reveal the mechanism of the regulatory role played by circFAM13B, miR-212 and E2F5 in HCC. This study provides a new theoretical basis and novel target for the clinical prevention and treatment of HCC.

## Supplementary Information

**Additional file 1: Table S1.** Primer sequences used for qPCR assays. **Table S2.** RNA probes for FISH.

## Data Availability

The circRNA expression data for HCC from GEO (https://www.ncbi.nlm.nih.gov/geo/query/acc.cgi?acc=GSE97332). The authors confirm that the data supporting the findings of the present study are available within the article and its Additional files.
