# Supplementary material for: CircFAM13B promotes the proliferation of hepatocellular carcinoma by sponging miR-212, upregulating E2F5 expression and activating the P53 pathway
Source: Cancer Cell Int. 2021 Aug 4;21:410. doi: 10.1186/s12935-021-02120-6 (PMC8335894; doi:10.1186/s12935-021-02120-6)
Supplement: Supplementary file 1 — Additional file 1: Table S1. Primer sequences used for qPCR assays. Table S2. RNA probes for FISH. [file 12935_2021_2120_MOESM1_ESM.docx]

| Gene | Sequence of the primers |
| --- | --- |
| circFAM13B-Forward | 5'-TCTCCCATCAGCATCCTACCA-3' |
| circFAM13B-Reverse | 5'-GCTGTTCCACAGCTGCTCTA-3' |
| LEMD3-Forward | 5'-AACAAGACGCGGAACAGTAAT-3' |
| LEMD3-Reverse | 5'-GAGTCCGTAAGTAGGAGAGGTC-3' |
| SPPL3-Forward | 5'-CAGACCTACTCGTGGGCCTAT-3' |
| SPPL3-Reverse | 5'-ACAGAGCCTGGGTAGAGTCAA-3' |
| KLF7-Forward | 5'-TGCTCTCAGCTCCGTAAAGGT-3' |
| KLF7-Reverse | 5'-GCTCTGTCCACTCTTAACGGC-3' |
| E2F5-Forward | 5'-GGGCTGCTCACTACCAAGTTC-3' |
| E2F5-Reverse | 5'-CCTACACCTTTCCACTGGATACT-3' |
| RPP14-Forward | 5'-GTTGATGCCGCCTTACCTTTG-3' |
| RPP14-Reverse | 5'-TCCACAATTTGACAAGACCACTG-3' |
| KCNK2-Forward | 5'-TAAATCTGCCGCTCAGAACTCC-3' |
| KCNK2-Reverse | 5'-TCCAATGCTTTGAACACGGTG-3' |
| SOX5-Forward | 5'-GAACAACAGGTGCTTGATGGG-3' |
| SOX5-Reverse | 5'-GCCCTCGGGATTCCCTATAAAT-3' |
| SKAP2-Forward | 5'-TCAGGGGATAAGAGCACTGATT-3' |
| SKAP2-Reverse | 5'-ACATCACCACGCTTAAATGACAA-3' |
| GAPDH-Forward | 5'-ACAACTTTGGTATCGTGGAAGG-3' |
| GAPDH-Reverse | 5'-GCCATCACGCCACAGTTTC-3' |

Table S1 Primer sequences used for qPCR assays

Table S2 Primer sequences used for qPCR assays

| **Gene name** | **probe sequence** |
| --- | --- |
| circFAM13B | TTTTATGGGTCGTGGGTATAGA |
| miR-212 | GGCCGTGACTGGAGACTGTTA |
